# Competing particle attractee in liquid bridges

**DOI:** 10.1098/rsta.2022.0302

**Published:** 2023-04-17

**Authors:** Robert Parker, Paolo Capobianchi, Marcello Lappa

**Affiliations:** Department of Mechanical and Aerospace Engineering, University of Strathclyde, James Weir Building, 75 Montrose Street, Glasgow G1 1XJ, UK

**Keywords:** Marangoni flow, liquid bridge, hydrothermal wave, particle accumulation structures

## Abstract

Assuming the so-called particle accumulation structures (PAS) in liquid bridges as archetypal systems for the investigation of particle self-assembly phenomena in laminar time-periodic flows, an attempt is made here to disentangle the complex hierarchy of relationships existing between the multiplicity of the loci of aggregation (streamtubes which coexist in the physical space as competing attractee) and the particle structures effectively showing up. While the former depends on purely topological (fluid-dynamic) arguments, the influential factors driving the outcomes of the fluid–particle interaction seem to obey a much more complex logic, which makes the arrangement of particles different from realization to realization. Through numerical solution of the governing Eulerian and Lagrangian equations for liquid and mass transport, we show that for a fixed aspect ratio of the liquid bridge, particles can be gradually transferred from one streamtube to another as the Stokes number and/or the Marangoni number are varied. Moreover, ranges exist where these attractors compete resulting in overlapping or intertwined particle structures, some of which, characterized by a strong degree of asymmetry, have never been reported before.

This article is part of the theme issue 'New trends in pattern formation and nonlinear dynamics of extended systems'.

## 1. Introduction

The occurrence of the so-called thermal Marangoni (or thermocapillary) flow is an area that has been actively investigated since the 1950s to gain a further understanding on what causes this phenomenon and how fluid properties affect it in relation to its patterning behaviour and the related hierarchy of bifurcations.

These flows are produced any time a temperature gradient exists in a direction parallel to the interface between two immiscible liquids or a liquid and a gas. This thermal inhomogeneity can cause variations in the surface tension distribution along such an interface, which behave as tangential forces forcing the liquid to move from the regions where the surface tension is smaller toward those where it is larger. Owing to viscous effects, this surface motion is then propagated to the entire bulk of the liquid, thereby producing relatively extended rolls, or vortices, which are generally regarded as the physical manifestation of this type of convection.

These vortices can attain a steady, oscillatory or even turbulent state depending on the magnitude of the temperature gradient and other physical parameters that relate to the considered liquid and the extension and shape of the domain where the fluid is hosted. In particular, these behaviors have been largely studied in relation to the so-called *liquid bridge*, namely a small volume of liquid suspended between two coaxial parallel discs (as with this configuration the ratio of the area of the free surface to that of the solid walls can be maximized, thereby allowing better observation of fluid motion). This flow has been revealed by seeding solid particles (tracers) in the liquid and monitoring their evolution over relatively extended periods of time.

Notably, previous research has revealed the existence of various waves that break the system's initial axi-symmetry, travel in different directions and give rise to oscillatory states with different spatial properties (e.g. pulsating or rotating patterns, [1–6]).

Given the specific nature of this subject, in this introduction, we wish to remark or emphasize that a fascinating (and unexpected) side effect of all this research is the discovery that this type of convection can support particle *self-organization and self-assembly mechanisms in certain situations* [7–9]. In other words, it has been found that if the right conditions are met in terms of aspect ratio and volume of the liquid bridge, Marangoni number, size and density of the tracers, these can demix from the fluid and accumulate, forming involved, almost one-dimensional, closed loops or circuits, generally simply referred to as PAS, i.e. particle accumulation structures [10–33].

The arrangement of particles within each of these realizations typically results in aggregates or structures that differ from realization to realization. However, in general, these are somehow ‘quantized’, that is, only a discrete number of morphologies or topologies have been observed (and seem to be allowed). The problem has been addressed through a synergetic combination of experimental, numerical and (ensuing) theoretical work aiming to identify the critical links between the physical and geometrical properties of PAS (as they manifest in the physical space) and the role played by the carrier flow in inducing/supporting them.

Although these phenomena hide a still-not-fully understood competition of complex and diverse physical mechanisms that ultimately determine macroscopic dynamics and many questions have still to be addressed, some consensus exists in the literature that the underlying cause-and-effect relationships obey some well-defined principles, that is, particles are allowed to change their initial spacing because of the ‘compressible’ nature of their motion. Put simply, these formations may be regarded as another example of the category of phenomena originally placed in a relevant theoretical context by Haller & Sapsis [34] and Sapsis & Haller [35]. Because of the finite size and mass of particles, their velocity and that of the carrier flow display a mismatch, which makes the related mathematical (and physical) properties different. While the fluid velocity is solenoidal (which implies the fluid volume is conserved), that of particles does not satisfy this mathematical constraint. As a result, the volume occupied by a certain number of particles at a given time is not conserved and the spacing among them may change in time [36]. This may be regarded as the necessary pre-requisite for the existence of ‘sinks’ in the particle velocity field, i.e. specific loci where particles can accumulate (to be referred to as ‘attractors’ using terminology borrowed from the field that studies the dynamics of nonlinear systems).

The effective spatio-temporal nature of these attractors, however, is still tied to the topology of the carrier flow since, if the particle Stokes number is not too high, the departure of particle trajectories from those followed by the fluid is appreciable, but, relatively ‘limited’. In particular, several studies have clarified that, in the case of Marangoni flow in liquid bridges, relevant insights into the mechanisms responsible for particle accumulation can be obtained by filtering out time-dependent effects and concentrating on what happens in a reference system rotating at the same angular frequency of the hydrothermal wave, which is produced as a result of the instability of Marangoni flow. More specifically, by using this mathematical artifice, it has been discovered that ‘closed streamtubes’ exist in the fluid velocity field, which might represent the sought aforementioned ‘sinks’ of particles.

Remarkably, in most cases, the topology of these streamtubes aligns well with the observed morphology of the emerging particle structures. Moreover, it has been shown that different closed streamtubes can coexist in the same velocity field, thereby opening the door to a potential problem of spatially competing attractors (also known as ‘multiplicity of solutions', [37]). Another open question concerns the ability of the emerging particle circuits to display a departure from the symmetry of these streamtubes in certain ranges of the Marangoni number and particle Stokes number.

In general, a problem still requiring attention is the unsolved issue of tracking the relationship between the closed streamtubes, the fluid streamlines and the particle patterns effectively showing up in the physical space, that is, determining whether and how some attractors are preferred by particles (instead of other coexisting ones) when the abovementioned parameters fall in given ranges. The present work may be regarded as a further step along these lines.

## 2. Mathematical model and numerical method

### (a) The fluid phase

The fluid problem is governed by the Navier–Stokes equations for an incompressible flow, in which gravity is neglected, and by the energy equation. In dimensionless form, these can be written as

2.1 $$\begin{array}{rl} \nabla \cdot u & \;=0, \end{array}$$

2.2 $$\begin{array}{rl} \frac{Du}{Dt} & \;=-\nabla p+\nabla^{2}u, \end{array}$$

**Figure 1.**
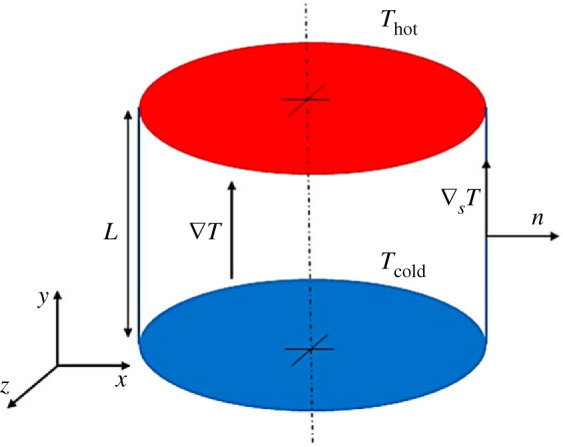
Schematic representation of the liquid bridge showing the temperature gradient and its projection along the interface (these two are coincident in this case as the interface is straight), and the unit vector perpendicular to the interface. (Online version in colour.)

2.3 $$\begin{array}{rl} \text{and}\;\;\frac{DT}{Dt} & \;=\frac{1}{\mathrm{Pr}}\nabla^{2}T, \end{array}$$

where u is the flow velocity vector, p is the pressure, T is the temperature and *L*, *ν*/ *L*, *ρν*^2^/ *L*^2^, *L*^2^/*ν* and Δ*T* have been used as reference quantities for the geometrical coordinates, velocity, pressure, time (*t*) and temperature, respectively (*L* being a characteristic length). The non-dimensional temperature is defined as $(T-T_{\text{ref}})/\Delta T$, where $T_{\text{ref}}$ is a suitable reference temperature (*T*_cold_ shown in figure 1 in our case). The operator D(⋅)/Dt is the usual material derivative, while Pr=ν/α is the Prandtl number, ratio between the fluid kinematic viscosity, ν, and the thermal diffusivity, α. This parameter is left unvaried throughout the whole study (Pr = 8, corresponding to NaNO_3_). Moreover, in line with the majority of existing efforts on the study of PAS, the physical properties of the fluid are assumed to be constant. Closure of the problem, however, requires the addition of the tangential stress conditions at the interface:

2.4 $$[\nabla u+{\left(\nabla u\right)}^{\text{T}}]\cdot n+\text{Re}\nabla_{\text{s}}T=0,$$

where n is the unit vector perpendicular to (pointing outward) to the interface, while $\nabla_{\text{s}}T$ is the projection of the temperature gradient on the surface separating the liquid bridge and the surrounding environment. It should be noted that the latter is assumed to be a gas characterized by a viscosity much smaller than that of the liquid bridge.

Accordingly, its contribution is disregarded in the tangential stress conditions and in equations (2.1–2.3), thereby allowing the description of the multiphase interfacial flow in the framework of a single-fluid approach. Moreover, heat exchange with the gas is also neglected and the free interface is modelled as a perfect cylinder. The former assumption is generally considered valid when the two supporting discs are heated and cooled ‘symmetrically’ with respect to the ambient temperature, i.e. their temperatures are *T*_a _+ Δ*T*/2 and *T*_a_ − Δ*T*/2, respectively, where *T*_a_ is the ambient temperature and Δ*T* is the overall temperature difference applied to the liquid bridge. In such circumstances, the temperature of the free surface (it is almost uniform with the exception of the changes that occur in proximity to the discs) is almost identical to that of the gas ambient, thereby minimizing the interfacial heat exchange. With regard to the latter hypothesis, the static curvature of the free interface can indeed be neglected as the liquid bridges are considered in microgravity conditions, their volume is identical to that of the corresponding cylinders having the same base and height and the wetting angle of the considered fluid is close to 90° (assuming the supporting discs to be coated with graphite, [8,38]). Dynamic shape deformations are also ignored by considering that the so-called Capillary number, defined as $\text{Ca}=\sigma_{\text{T}}\Delta T/\sigma_{\text{o}}$, is much smaller than 1 for the conditions considered in the present work ($\sigma_{\text{o}}$ being the reference surface tension evaluated at *T*_cold_, i.e. $\sigma_{\text{o}}≅1.15\times 10^{-1}\text{N}{\text{m}}^{-1}$; $\sigma_{\text{T}}≅7\times 10^{-5}\text{N}{\text{K}}^{-1}{\text{m}}^{-1}$ → $\text{Ca}≅6\times 10^{-4}\;\Delta T$). The dimensionless parameter $\text{Re}=\sigma_{T}\Delta TL/\rho \nu^{2}$ appearing in equation (2.4) is the Reynolds number based on the characteristic thermocapillary velocity $U_{\text{T}}=\sigma_{\text{T}}\Delta T/\rho \nu$, where $\sigma_{T}=-\partial \sigma /\partial T$ is the derivative of the surface tension σ with respect to the temperature, ΔT is the aforementioned temperature difference between the two cylindrical rods supporting the liquid bridge, and *L* is the distance between them. It is usual practice to refer to the Marangoni number Ma=RePr rather than to the Reynolds number to characterize the flow field, therefore, in the following this parameter is used instead of Re to describe the results.

The additional thermal and kinematic boundary conditions for the fluid phase schematically shown in figure 1 can be turned into precise mathematical relationships as follows:

On the two supporting discs:

2.5 $$\begin{array}{rl} & \text{Cold disc }(y=0):\;T=0,\;\;u=0. \end{array}$$

2.6 $$\begin{array}{rl} & \text{Hot disc }(y=1):T=1,\;\;u=0. \end{array}$$

At the free surface:

2.7 $$\begin{array}{rl} & \partial T/\partial n=0\;\;(\text{adiabatic}\;\text{behaviour}). \end{array}$$

2.8 $$\begin{array}{rl} & u\cdot n=0\;\;\text{( no}\;\text{radial}\;\text{velocity) }. \end{array}$$

### (b) The solid phase

Particles are tracked individually in a Lagrangian manner. Owing to their small diameter and small particle-to-fluid volume fractions usually considered in these problems (see [8], for instance), the back influence of the particles on the flow field and mutual particle interactions can be neglected. This makes a simple one-way coupling strategy for particle tracking ideally suited to address the considered problem. The Saffman lift force, the Faxén corrections and the influence of the Basset force can also be disregarded (see, e.g. [22,39]). In such a simplified theoretical framework, the particle motion is finally described by the following, reduced, Maxey-Riley equation (refer, e.g. to the work of [40])

2.9 $$\frac{\text{d}v}{\text{d}t}=\frac{1}{\xi +1/2}\left[\frac{3}{2}\frac{\text{D}u}{\text{D}t}-\frac{\left(v-u\right)}{St}\right],$$

and

2.10 $$\frac{\text{d}x}{\text{d}t}=v,$$

where v is the particle velocity calculated from the Lagrangian coordinate x, $\xi =\rho_{\text{p}}/\rho$ is the ratio between the particle density, $\rho_{\text{p}}$, and the fluid density, ρ, and $St=d_{\text{p}}^{2}/\text{18}L^{2}$ is the Stokes number in which $d_{\text{p}}$ represents the diameter of the particle and *L* is the height of the liquid bridge.

The boundary conditions for the fluid phase reported in §2(a) are naturally complemented by those considered for the particles. These are set here in the framework of the so-called *local interaction model* (more details can be found in [32]) by which the component of the particle velocity perpendicular to the wall is annihilated as the particle approaches the boundary while its tangential velocity is kept unchanged. This artifice is instrumental in ensuring that (finite-size) particles are prevented from violating the physical impenetrability of solid walls. As due to lubrication effects, a similar condition also holds for the free surface, the same model is also used at the liquid/gas interface.

### (c) The numerical framework

Simulations have been carried out adopting the ‘buoyantBoussinesqPimpleFoam’ transient solver available in OpenFOAM, properly complemented by the thermocapillary stress condition equation (2.4) (see e.g. [33,37]). In the present case, obviously, the acceleration of gravity has been set to zero (microgravity conditions), which means that only the fluid-volume preserving abilities of the solver have been exploited (incompressible flow).

In OpenfFOAM, the governing equations for the fluid phase (i.e. equations 2.1–2.3 in our case) are discretized using the Finite Volume Method (FVM). Moreover, the solver relies on the PISO algorithm of Issa [41], where a collocated grid arrangement of the variables is used to integrate the momentum equations and enforce mass conservation. The energy equation (2.3) is subsequently solved in a segregated manner. Integration in time of both the thermo-flow field and the particles governing equation is based on the backward Euler scheme. Convective terms have been discretized using the second-order accurate, linear central-differences scheme.

All these kernels have already been used in the previous studies by Capobianchi and Lappa [32,33,37] to which the interested reader is referred for additional details about the numerical implementation. Here, we wish simply to recall that Capobianchi & Lappa [32] provided evidence for the reliability and accuracy of these kernels through focused comparison with the simulations by Melnikov *et al*. [13], and the independent numerical study by Lappa [22]. Excellent agreement was obtained in terms of fundamental properties of the supercritical Marangoni flow (the frequency of the hydrothermal wave for a liquid bridge with aspect ratio (height/diameter) *A *= 0.34 and Ma = 20 600) and the morphology of the emerging particle structures (for ξ=1.85 and *St *= 10^−4^).

As a concluding remark for this section, we wish to point out that all the numerical results presented in §3 have been obtained using a mesh having the *M*_1_ resolution defined in the earlier study by Capobianchi & Lappa [32] for the same value of the Prandtl number considered here (i.e. Pr = 8). Such a resolution was found to provide a good compromise between computational times and accuracy over an extended range of values of the Marangoni number (see Table III in [32]). The corresponding values taken by the ratio of the maximum particle diameter over the minimum computational-cell size for the conditions examined in §3 (particle Stokes number between $5.3\times 10^{-6}\leq St\leq \text{8.5}\times {\text{10}}^{-5}$ and $6\times 10^{-7}\leq St\leq \text{3.9}\times {\text{10}}^{-5}$ for *A *= 0.34 and *A *= 0.5, respectively) are less than 1 for all the considered circumstances, the only exception being the particle with diameter 80 µm in the *A *= 0.34 case (for which the particle-to-cell ratio slightly exceeds the unit value, which however we still consider acceptable given the one-way nature of the particle-fluid coupling implemented here).

## 3. Results

### (a) Hydrothermal wave

**Figure 2.**
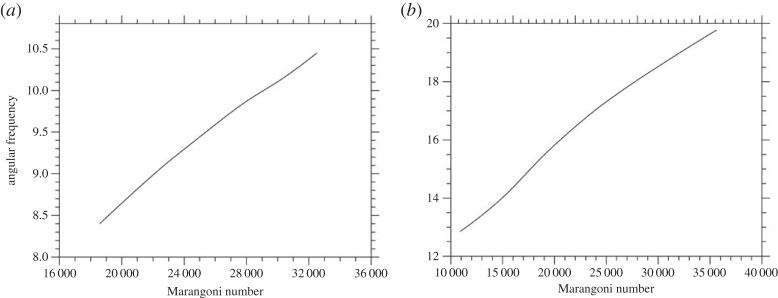
Non-dimensional angular frequency (made non-dimensional using *L*^2^/*ν* as reference time) as a function of the Marangoni number: (*a*) *A *= 0.34, (*b*) *A *= 0.5.

As a prerequisite for the computation of PAS, the angular frequency of the travelling wave (defined as 2πf/m where *f* is the frequency in Hz) has been determined over the entire range of considered values of the Marangoni number. The outcomes of this initial study are summarized in figure 2*a,b* for the liquid bridge with aspect ratio 0.34 and 0.5, respectively.

**Figure 3.**
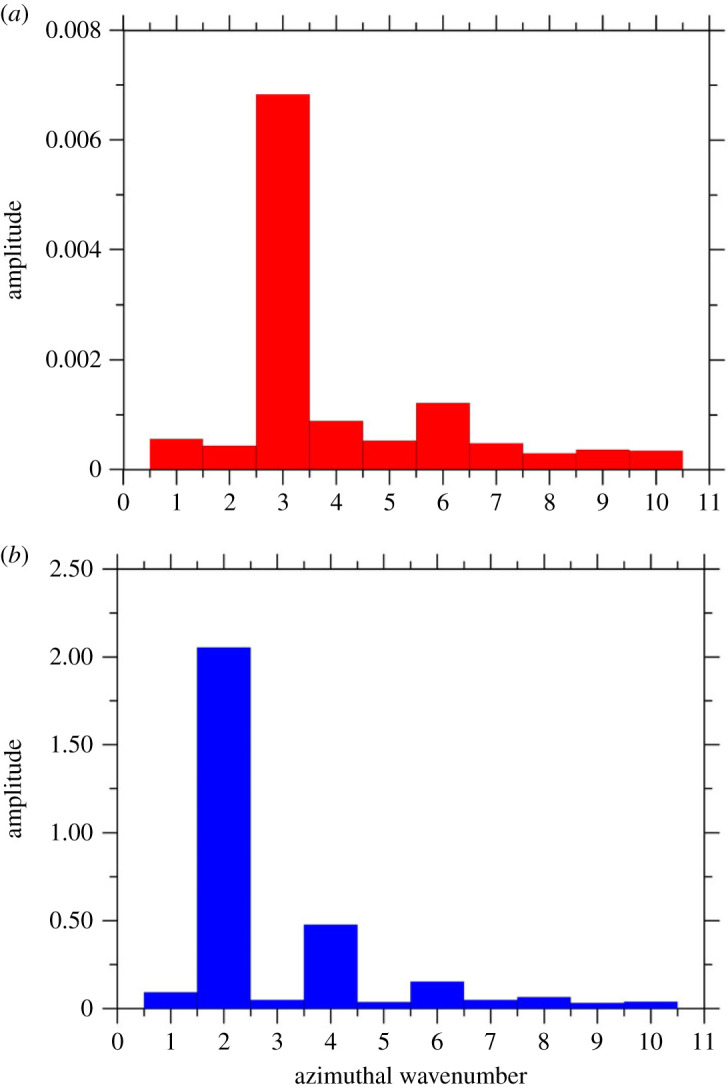
Spectrum of the surface temperature distribution (surface temperature profile taken for y=L/2): for (*a*) *A *= 0.34 and Ma = 32 500, (*b*) *A *= 0.5 and Ma = 35 600. (Online version in colour.)

As the reader will realize by inspecting these two figures, the angular frequency behaves as an increasing function of the Marangoni number. The corresponding (spatial) spectrum of the surface temperature distribution (amplitude versus azimuthal wavenumber *m*) for a couple of representative cases is shown in figure 3*a,b* (yet for *A *= 0.34 and *A *= 0.5, respectively). The major outcome of these figures resides in their ability to make evident that the increase in the aspect ratio from *A *= 0.34 to *A *= 0.5 essentially causes a switch from m=3 as the dominant wavenumber (the multiple m=6 being also present for relatively high values of the Marangoni number, figure 3*a*) to *m *= 2. For relatively high values of the Marangoni number, the related multiples *m *= 4 and *m *= 6 also pop up in the spectrum (figure 3*b*).

### (b) PAS classification

Owing to space limitations, in this section we do not strive to describe all the known details related to the morphology of PAS as these are available in relevant studies published over the last 10 years (the reader being referred, e.g. to [7,11,12,16,21–23,26,30,31,42]). Rather, we concentrate on those aspects which, in our opinion, may help to put the present work under the right perspective, that is, identifying the complex relationship among the emerging properties of these phenomena, ‘environmental’ factors (namely, the topology of the carrier flow) and the nature of the dispersed phase *per se* (density and diameter of the spherical particles). Therefore, we recall some existing ‘classifications’ only because they can make the above process more ‘rational’ and help the reader to place the present findings in the right theoretical context in the light of earlier studies on the subject.

In this regard, an intrinsic property of PAS playing an important role in the sought hierarchy of relationships is certainly the so-called number of loops, *N*, i.e. how many times the one-dimensional structure formed by the particles wounds about the vortex core. This number is known to be an integer multiple of the azimuthal wavenumber *m* (*N *= *m* or *N *= 2*m*), which explains why a dichotomy exists in the literature about two distinct families of PAS, generally known as SL-I [11,12] or SL-II [7,16]. Additional notions and definitions, such as the linear extension of the PAS, its inner core radius, and the area of the ‘petals’ or ‘blades’, have been introduced over the years to allow a precise quantification of a series of purely geometrical effects [26,32,42]. Another distinguishing mark of these structures is their ability to transcend the intrinsic symmetries of the carrier flow, i.e. to show up with shapes that are not a trivial manifestation of the underlying attractors in the velocity field (the aforementioned closed streamlines or tubes in the reference system rotating at the same angular velocity of the hydrothermal wave).

Given these premises, we therefore follow these earlier classifications to map the emerging PAS structures into the corresponding basin of attraction in the space of parameters (aspect ratio, Marangoni number, particle density ratio and Stokes number). In particular, besides considering two different aspect ratios (*A *= 0.34 and *A *= 0.5), the following cases are explored: Marangoni number spanning the interval from 10^4^ to 3.5 × 10^4^, particle Stokes number between $5.3\times 10^{-6}\leq St\leq \text{8.5}\times {\text{10}}^{-5}$ and $6\times 10^{-7}\leq St\leq \text{3.9}\times {\text{10}}^{-5}$ for *A *= 0.34 and *A *= 0.5, respectively, and density ratio fixed to *ξ*=1.85.

It is shown that the related tree of relationships is often counterintuitive and (in line with the findings by other authors) often displays a non-monotonic behaviour. Along these lines, some effort is provided here to show that this is the rule rather than the exception and that the ‘multiplicity of solutions' seems to be an intrinsic feature of this class of phenomena. This is indeed the key we use to interpret their ‘scattered’ appearance in the space of parameters and the coexistence (in the physical space) of structures with different geometrical properties (see §4).

### (c) Shallow liquid bridge

For simplicity we start from the liquid bridge with an aspect ratio *A *= 0.34 as most of the results reported in the literature concern this specific case.

**Figure 4.**
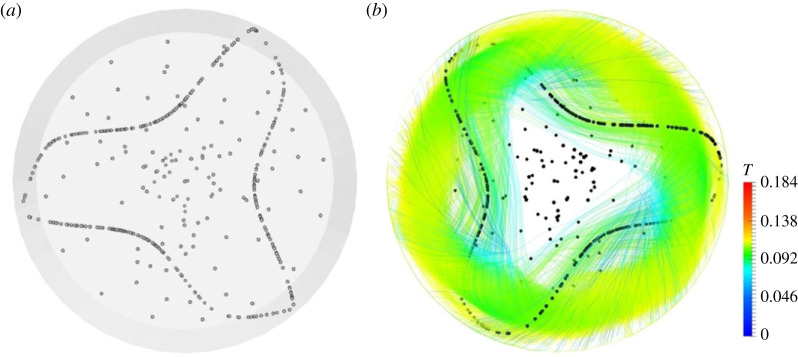
PAS (SL-I) for Ma = 28 000 and particle diameter of 80 µm (t≅60): (*a*) top view, (*b*) view from below of PAS and streamlines coloured according to the corresponding temperature distribution. (Online version in colour.)

As the reader will realize by taking a look at table 1, for this value of the aspect ratio the most common structure revealed by the numerical simulations is the classical Single Loop 1 (SL-I), see, e.g. figure 4*a* for Ma = 28 000 and $St≅\text{8.5}\times {\text{10}}^{-5}$.

**Table 1.** Map of PAS states found for the liquid bridge with aspect ratio *A *= 0.34 as a function of the Marangoni and Stokes numbers.

		PAS type versus Marangoni number						
$d_{p}(\mu m)$	Stokes number	18 600	20 460	23 000	25 500	28 000	30 000	32 500
20	$5.340\times 10^{-6}$	—	SL-I	—	—	—	—	—
32	$1.367\times 10^{-5}$	no	U1	no	no	SL-I	Y	no
40	$2.136\times 10^{-5}$	—	SL-I	—	—	—	—	—
45	$2.703\times 10^{-5}$	Y	SL-I	No	SL-I	SL-I	Y	U2
60	$4.806\times 10^{-5}$	Y	SL-I	No	SL-I	SL-I	Y	Y
80	$8.544\times 10^{-5}$	no	SL-I	SL-I	ASL-I	SL-I	SL-I	SL-I

As made evident by this figure, for *m *= 3, the classical SL-I topology simply consists of a single circuit with the presence of three ‘blades’ wrapped around the Marangoni toroidal vortex, which mimic almost perfectly a $T_{3}^{3}$ streamtube existing in the base flow field [43]. In this regard, it is worth recalling that a relevant classification of these streamtubes was originally introduced by Kuhlmann & Muldoon [17]. By using a relevant synthetic flow, these authors could show that different closed streamtubes of relatively large size exist in *m* = 3 flows, namely: $T_{3}^{3}$, $T_{3}^{6}$ and three streamtubes of the $T_{1}^{3}$ type, shifted by 2π/3 relative to one another (in such analysis, streamtubes were classified as $T_{i}^{j}$ where *T* indicates a closed streamtube of period *i* that is *j* times wrapped about the basic toroidal vortex).

The present $T_{3}^{3}$ -type PAS shown in figure 4*a* (seen from above) is complemented by the corresponding from-below view in figure 4*b*, which provides a good impression of the associated three-dimensional fluid motion.

**Figure 5.**
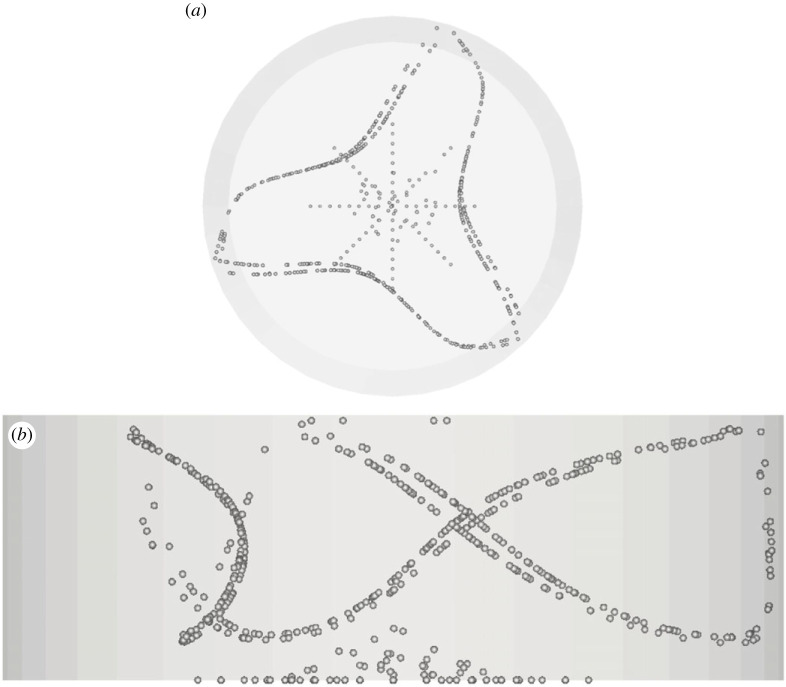
PAS (ASL-I) for Ma = 25 500 and particle diameter 80 µm (t≅25): (*a*) top view, (*b*) side view.

Departures from this condition, however, are possible. As a first case of alternate PAS realization, the reader may consider for instance the asymmetric structure (Asymmetric Single Loop 1 (ASL-I)) emerging for the same value of the particle Stokes number but a slightly smaller Marangoni number (Ma = 25 500, figure 5). In this case, the PAS shows up as a series of loops slightly offset from each other.

**Figure 6.**
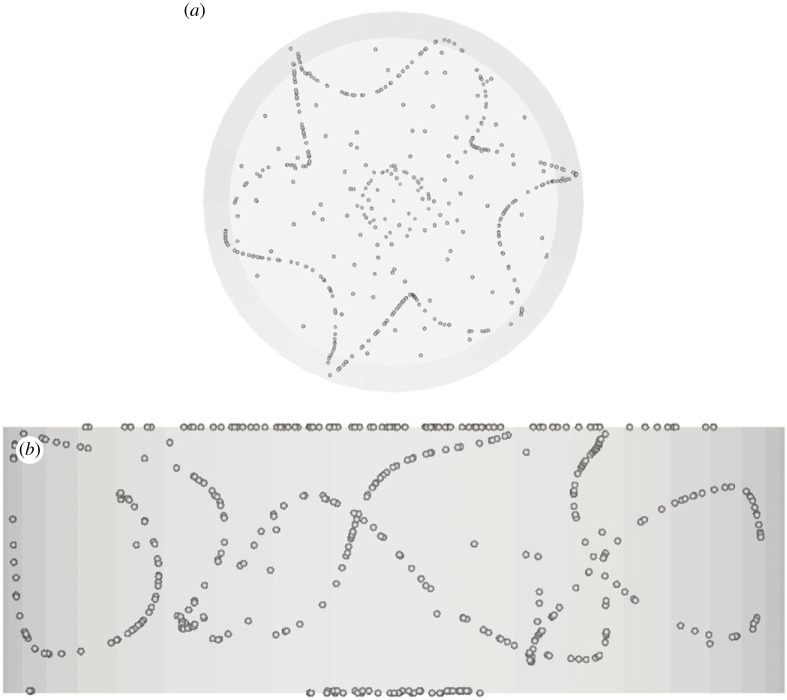
PAS (Y) for Ma = 30 000 and particle diameter of 32 µm (t≅104, *N*_cr_* *= 167): (*a*) top view, (*b*) side view.

The most interesting variants, however, are obtained as the Stokes number is decreased. A particle circuit with six loops, indeed, emerges as St becomes smaller than $4.8\times 10^{-5}$ (figure 6). Interestingly this specific realization does not represent a member of the SL-II family and should rather be regarded as the manifestation of the different attractor $T_{3}^{6}$ coexisting in the physical space with the aforementioned $T_{3}^{3}$ streamtube. Although, particle structures (denoted by the Y symbol in table 1) driven by this attractee have been found to be ‘unstable’ in earlier studies on this subject [43], the present simulations show that this streamtube can become a stable locus of accumulation for specific combinations of the Marangoni and particle Stokes number (see again table 1). In particular, this formation exists for Ma = 18 600, Ma = 30 000 and Ma = 32 500, provided St is not higher than 4.8 × 10^−5^ and not smaller than 1.37 × 10^−5^, suggesting that particles show a preference for different attractors outside this range. In order to verify its stability, the related simulations have been prolonged over a non-dimensional time exceeding *t *= 100 (corresponding to $N_{\text{cr}}≅160$ complete revolutions of the travelling wave for Ma = 30 000; for larger Ma, i.e. Ma = 32 500, *t* and *N*_cr_ have been extended up to 400 and 660, respectively).

**Figure 7.**
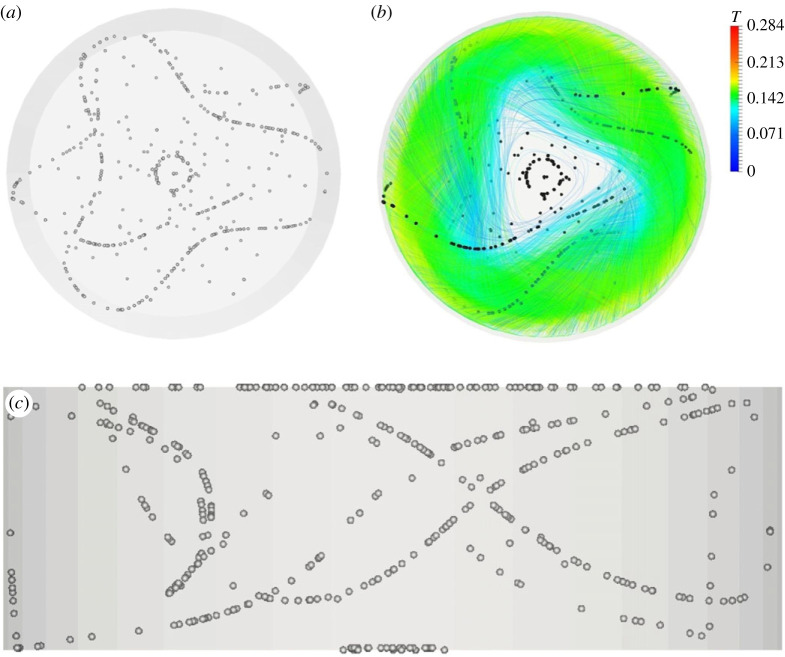
PAS (U1) for Ma = 20 460 and particle diameter of 32 µm (t≅414, *N*_cr_* *= 575): (*a*) top view, (*b*) view from below of PAS and streamlines coloured according to the corresponding temperature distribution, (*c*) side view. (Online version in colour.)

**Figure 8.**
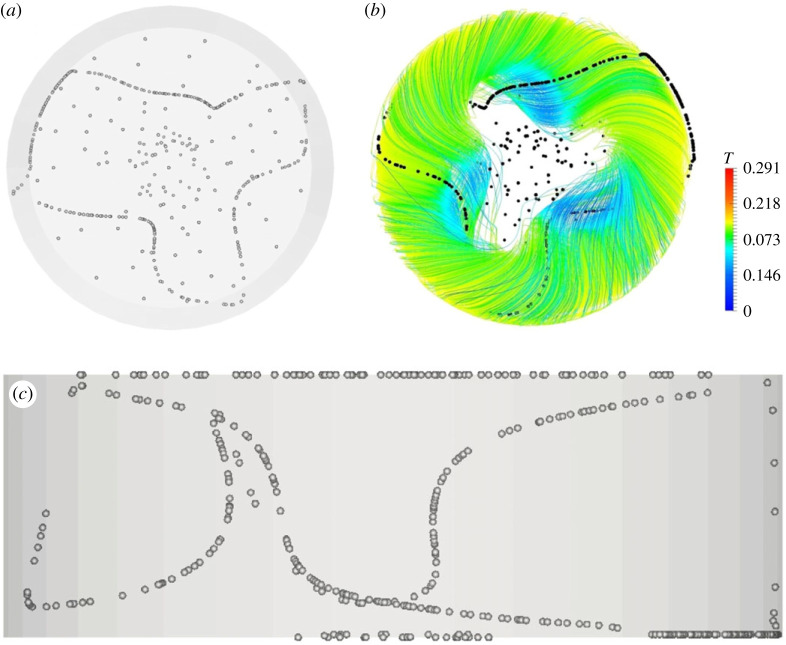
PAS (U2) for Ma = 32 500 and particle diameter of 45 µm (t≅85): (*a*) top view, (*b*) view from below of PAS and streamlines coloured according to the corresponding temperature distribution, (*c*) side view. (Online version in colour.)

The most interesting outcomes of this parametric study, however, concern the identification of two completely new structures, namely, those shown in figures 7 and 8.

The classical 3-lobe PAS can still be recognized in figure 7 for Ma = 20 460 and $St≅\text{1.37}\times {\text{10}}^{-5}$, which indicates that the $T_{3}^{3}$ streamtube still plays the role of template for the accumulation of particles for this specific combinations of Ma and *St*. The significance of this figure, however, resides in the evidence it provides for the tendency of particles to gather on two different (spatially separated) attractors at the same time, which indicates that specific conditions exist for which coexisting streamtubes can ‘compete’ and produce independent (although intertwined in space) particle circuits. Apart from the interesting implications in terms of ‘multiplicity’ of PAS solutions that attaches to this observation (we will return to this interesting concept later), this figure also shows for the very first time that particles can also select a streamtube of the $T_{1}^{3}$ type (predicted by [17]) for which no related PAS have been reported in the literature so far. To verify the stability of this coexistence, this simulation has been conducted over a non-dimensional time as high as *t *= 400 (corresponding to more than 500 complete revolutions of the travelling wave for this value of the Marangoni number).

Finally, figure 8 may be regarded as a notable example of conditions for which the particle circuit (yet of the SL-I type) displays a remarkable asymmetry in the size of the three lobes (labelled as U2 state in table 1).

### (d) Intermediate aspect ratio liquid bridge

Having completed a description of the emerging particle patterning behaviour as a function of the Marangoni and Stokes number for *A *= 0.34 for which the dominant azimuthal wavenumber is *m *= 3 over the entire interval of considered Marangoni numbers, we turn now to the case *A *= 0.5 for which the dominant wavenumber is *m *= 2. Following the same approach undertaken in the earlier section, all the results are summarized synthetically in a single table by which the topology of the emerging structures can be mapped into the corresponding space of parameters (table 2).

**Table 2.** Map of PAS states found for the liquid bridge with aspect ratio *A *= 0.5 as a function of the Marangoni and particle Stokes numbers.

		PAS type versus Marangoni number								
$d_{p}(\mu m)$	Stokes number	10 900	12 700	15 210	16 400	19 100	23 960	27 400	31 460	35 600
10	$6.173\times 10^{-7}$	no	no	SL-II	no	SL-II	ASL-I	ASL-I	SL-I	SL-I
25	$3.858\times 10^{-6}$	no	no	SL-II	no	ASL-II	ASL-I	ASL-I	SL-I	SL-I
32	$6.321\times 10^{-6}$	no	no	SL-II	no	SL-II	ASL-I	ASL-I	SL-I	SL-I
50	$1.543\times 10^{-5}$	no	no	SL-II	no	SL-II	ASL-I	ASL-I + SL-II	SL-I + ASL-II	SL-I + ASL-II
80	$3.951\times 10^{-5}$	no	no	ASL-II	no	ASL-II	SL-I	SL-I + SL-II	SL-I + ASL-II	SL-I

**Figure 9.**
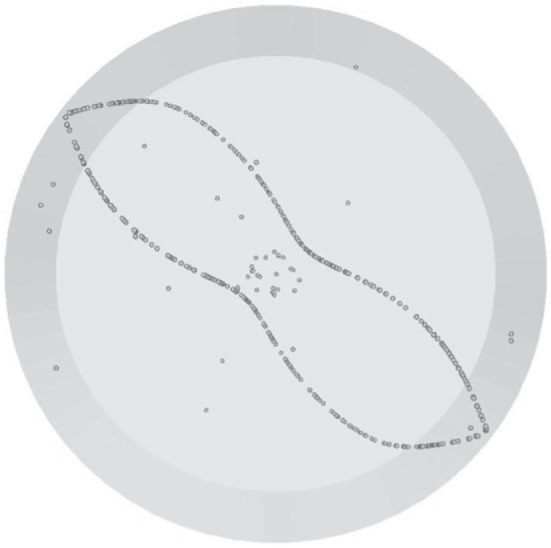
Top view of PAS (SL-I) for Ma = 35 600 and particle diameter of 80 µm (t≅9).

As a fleeting glimpse into this table would confirm, classical structures of the SL-I type are still possible in this case, although they can be found essentially in the high Marangoni number range, i.e. for Ma = 31 460 and Ma = 35 600 (e.g. figure 9).

**Figure 10.**
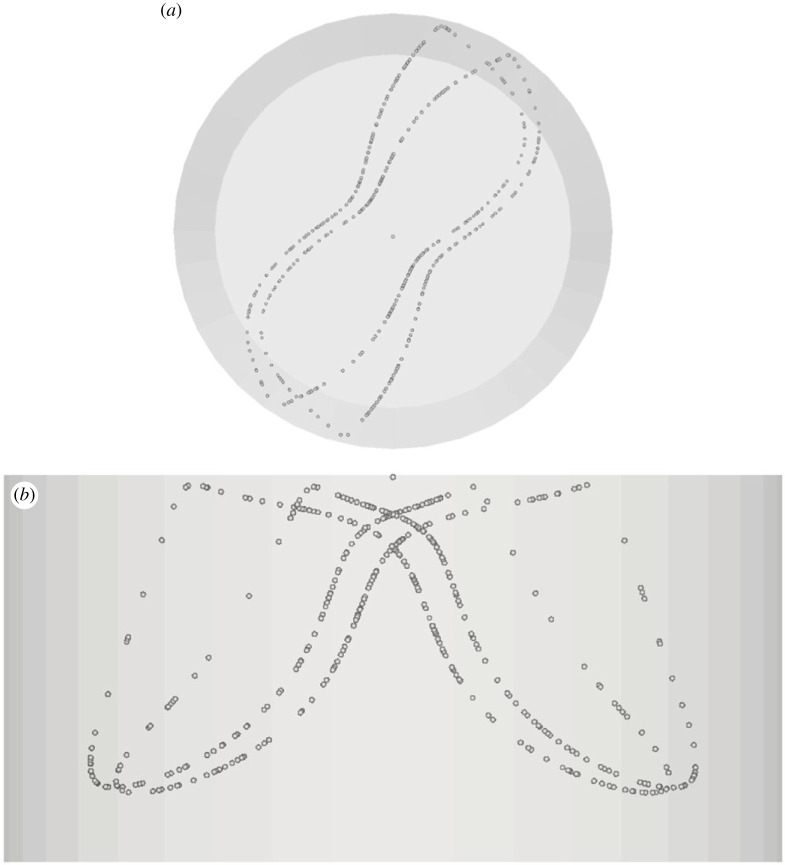
PAS (ASL-I) for Ma = 23 960 and particle diameter of 32 µm (t≅42): (*a*) top view, (*b*) side view.

On decreasing the Marangoni number to Ma = 23 960, asymmetric single-loop structures become the dominant PAS type (e.g. figure 10).

Closer inspection of table 2, however, reveals that unlike the *m *= 3 case, formations of the SL-II type are relatively common for this aspect ratio over large ranges of the particle Stokes number if relatively small values of the Marangoni number are considered (Ma = 19 100 and Ma = 15 210).

**Figure 11.**
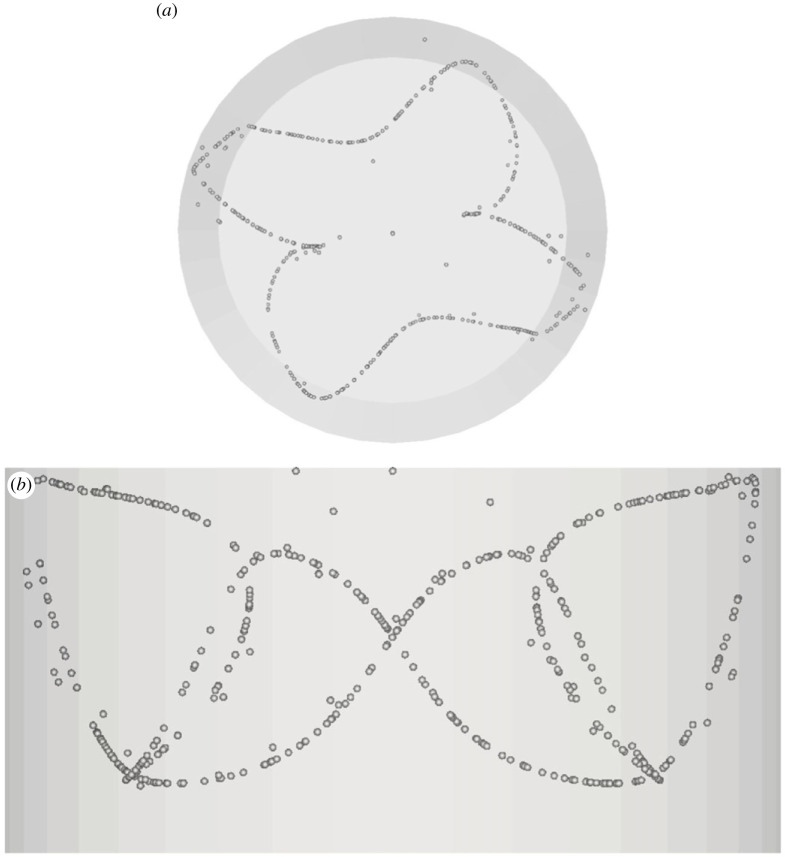
PAS (SL-II) for Ma = 19 100 and particle diameter of 50 µm (t≅9): (*a*) top view, (*b*) side view.

An example of this patterning behaviour, closely resembling that reported by other authors (see, e.g. [18,28]) can be seen in figure 11.

**Figure 12.**
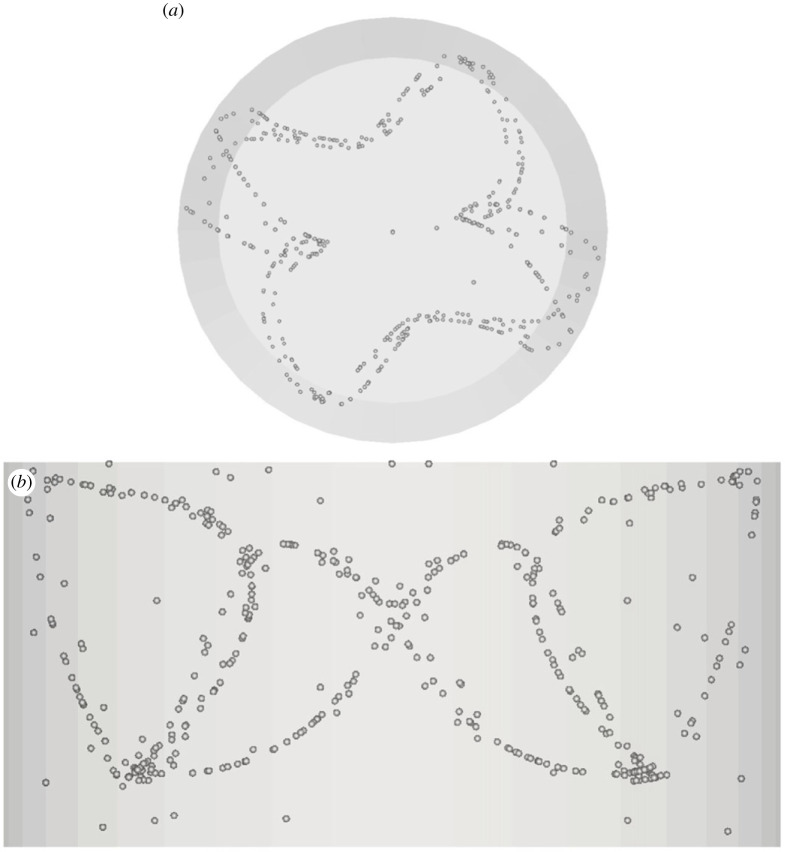
PAS (ASL-II) for Ma = 19 100 and particle diameter of 25 µm (t≅13): (*a*) top view, (*b*) side view.

Additional insights into the evolutionary progress of these formations as the particle Stokes number is varied follow naturally from a comparison of figures 11 and 12. By visual inspection of the latter, the reader will realize that, on decreasing the Stokes number for a fixed Ma, the symmetric double-loop circuit can be turned into a heretofore unseen (not reported in the literature until now) asymmetric PAS variant (ASL-II).

**Figure 13.**
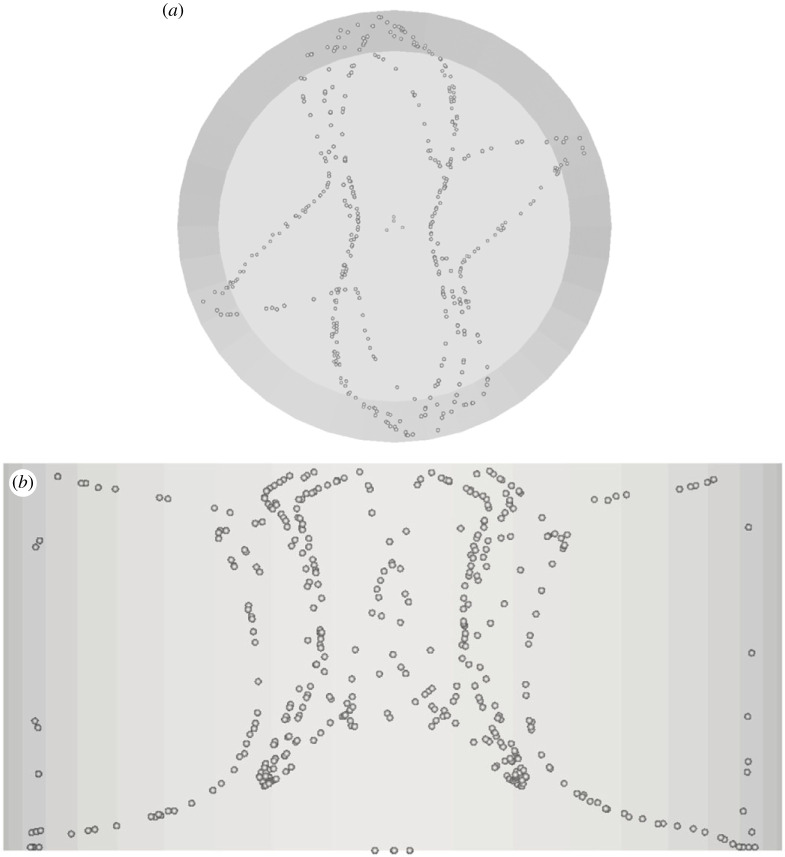
PAS (ASL-I + SL-II) for Ma = 27 400 and particle diameter of 50 µm $\left(t≅50,N_{\text{cr}}≅150\right)$: (*a*) top view, (*b*) side view.

**Figure 14.**
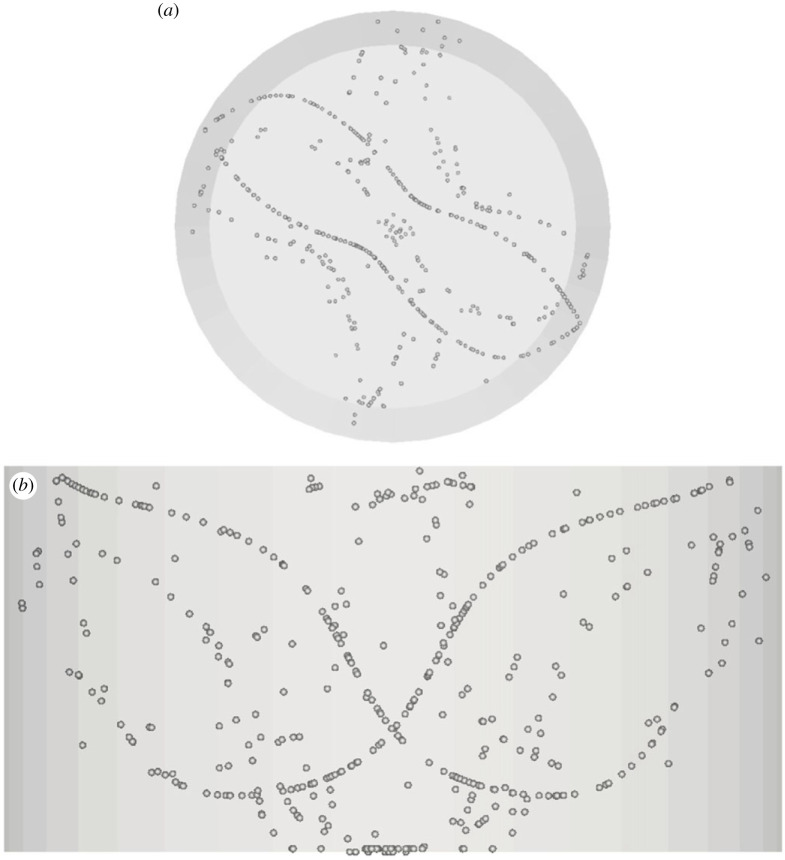
PAS (SL-I + ASL-II) for Ma = 31 460 and particle diameter of 50 µm $\left(t≅50,N_{\text{cr}}≅170\right)$: (*a*) top view, (*b*) side view.

**Figure 15.**
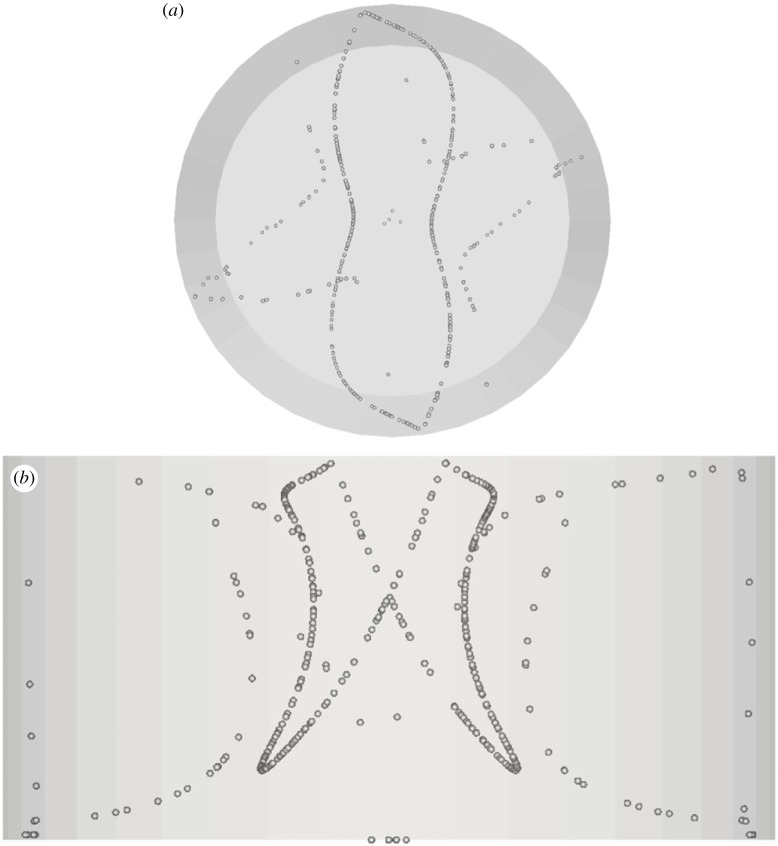
Top view of PAS (SL-I + SL-II) for Ma = 27 400 and particle diameter of 80 µm $\left(t≅50,N_{\text{cr}}≅150\right)$: (*a*) top view, (*b*) side view.

The next figures of the sequence (figures 13–15) simply illustrate that the ability to support coexisting particle circuits (as discussed in §3(c) and shown in figure 7) is not an exclusive prerogative of the *m *= 3 mode. The *A *= 0.5 aspect ratio can also give rise to unusual realizations if relatively large values of the Stokes number are considered in the high-Ma range of the space of parameters. These figures are instrumental in showing that the SL-I and SL-II can coexist, displaying various combinations in terms of symmetric or asymmetric variants. In line with the strategy already implemented in §3(c), in order to assess the stability of this coexistence, a longer non-dimensional time has been simulated (exceeding 100 complete revolutions of the travelling wave for all these cases).

## 4. Discussion and conclusion

A thorough interpretation of the phenomena presented in the preceding section is obviously not possible given the level of knowledge available on such a subject, which is still relatively limited. Nevertheless, in the following an attempt is made to create the right connections with available theories or models and to infer general behaviours through a critical review of other experimental or numerical results appearing in the literature.

We have already reported at the beginning of §3(c) the classification introduced by Kuhlmann and coworkers in terms of topology and multiplicity of the aforementioned streamtubes (the closed tubes that a single toroidal roll can support in the reference system rotating at the same angular velocity of the hydrothermal wave, which Kuhlmann & Muldoon [17] synthetically categorized as $T_{i}^{j}$ where *T* indicates a closed streamtube of period *i* that is *j* times wrapped about the basic toroidal vortex).

It is also worth recalling that later experimental and numerical studies for real flows have shown that only a subset of these $T_{i}^{j}$ attractors seem to effectively support the formation of particle structures. As an example, while PAS closely mimicking the topology of the $T_{3}^{3}$ have been found frequently, only a handful of results are available about structures resembling the $T_{3}^{6}$. Moreover, these 6-lobe particle circuits have generally been observed as transient phenomena in the process leading to the classical 3-lobe configuration or as structures coexisting with the standard 3-lobe configuration [19]. It should also be pointed out that, before the present study (figure 7), no results were available at all with regard to PAS driven by the $T_{1}^{3}$ attractor.

For the case *m *= 3, interesting experimental results are due to Toyama *et al*. [42], who considered shallow liquid bridges (0.32 ≤ *A* ≤ 0.34) made of 2 cSt silicone oil with Pr≅35.6 and particles with density ratio *ξ* = 2. For a fixed particle diameter (corresponding to $St=\text{4.3}\times {\text{10}}^{-6}$, $St=\text{4.6}\times {\text{10}}^{-6}$ and $St=\text{4.9}\times {\text{10}}^{-6}$ for *A *= 0.32, *A *= 0.33 and *A *= 0.34, respectively), they found that a small increase in the aspect ratio for a fixed Marangoni number or an increase in Marangoni number for fixed aspect ratio can cause a transition from SL-I to SL-II passing through the intermediate state where these structures overlap (in particular, while for *A *= 0.32, the SL-I is dominant, for *A *= 0.34 this role is taken on by the SL-II).

For *m *= 2 a relatively wide database of experimental results can be found in Gotoda *et al*. [27]. Using liquid bridges of decane (C_10_H_22_, Pr≅13.5) with aspect ratio *A *= 0.45, $3\times 10^{4}<\text{Ma}<6\times 10^{4}$, particles with various values of the Stokes number $7\times 10^{-9}\leq St\leq \text{4.5}\times {\text{10}}^{-5}$ and density ratio 0.95≤ξ≤4.78, these authors identified a strong connection between the emerging PAS type and the Marangoni number (the SL-I PAS being taken over by SL-II as the Marangoni number exceeds a given threshold).

For the same liquid and aspect ratio, through numerical simulations, Melnikov & Shevtsova [28] revealed an interesting sequence of changes in the topology and symmetry of the emerging PAS on increasing the particle Stokes number *at a fixed value of the Marangoni number* (Ma = 3.8 × 10^4^) and a fixed density ratio *ξ*=1.01 (almost isodense fluid and particles). Yet for m=2, they observed no PAS for $St=\text{5}\times {\text{10}}^{-6}$, a first regime with ASL-I for $5.1\times 10^{-9}<St<\text{1.4}\times {\text{10}}^{-5}$, coexistence of SL-I and SL-II for $1.4\times 10^{-5}<St<\text{2.3}\times {\text{10}}^{-5}$, SL-I for $2.3\times 10^{-5}<St\leq \text{3}\times {\text{10}}^{-5}$ and again ASL-I for $3.15\times 10^{-5}<St\leq \text{4.5}\times {\text{10}}^{-5}$. As a mark distinguishing the two regimes of ASL-I lying at the two opposite ends of the explored interval of Marangoni numbers, these authors invoked a slightly different topological behaviour (consisting in the branches of the structures partly overlapping or only crossing, respectively).

These studies considered different fluids, therefore making a direct comparison among them or with the present findings impossible. Anyhow, we wish to remark that, although all these results are quite scattered in the space of parameters and identifying universality classes is quite difficult, some general trends or commonalities can be distilled out. Indeed, collectively, they confirm that ‘intermittent’ behaviours with alternating intervals of existence and non-existence, or PAS of the SL-I type being taken over by the SL-II or vice-versa as the governing parameters change, are quite common, and the findings presented in §3 are not an exception to this rule. Taken together, all these aspects or observations should be regarded as proof or demonstration of the high sensitivity that these phenomena exhibit to the physical properties of the considered particles. Another way to think about this concept is to consider that the existence of closed streamtubes (the aforementioned fluid-dynamic attractors) is a condition *necessary*, but *not sufficient* for the manifestation of particle structures. The finite size or mass of the particles is the additional ingredient determining whether the particle will undergo accumulation, or not, and which attractor they will effectively select.

Given the paucity of available data, providing only disjoint glimpses of qualitatively and quantitatively different results in different parts of the parameter space, it cannot be excluded that many investigators could not observe additional PAS variants (some of which have been revealed by the present study) simply because they exist in narrow intervals of the Marangoni and Stokes numbers and their explorative efforts did not ‘intersect’ these ranges of existence.

For the circumstances considered in the present work, while for *m *= 3 single loop structures are favoured over the entire range of Marangoni and Stokes numbers examined, for *m *= 2 double-loops become the preferred PAS for intermediate values of the Marangoni number (with the SL-I being recovered in the high-Ma range, eventually coexisting with the SL-II for relatively high values of the Stokes number). In both cases, occasional departures from classical PAS are possible in the form of circuits de-centered with respect to the symmetry axis of the liquid bridge or more *exotic variants*, which should be regarded as the major novelty or contribution of the present work to the existing literature. We refer in particular to the exemplars shown in figures 6–8 and 12.

The major significance of figure 6 resides in the evidence it provides about the effective possibility to obtain 6-lobe structures as a stable independent state in certain ranges of the Stokes number and for specific values of the Marangoni number. Along the same lines, figure 9 is also extremely interesting as, for the first time, a configuration has been obtained where a classical well-formed 3-lobe PAS coexists with an independent particle circuit corresponding to one of the aforementioned $T_{1}^{3}$ attractors (another key observation concerning this specific realization is that it should be considered relatively ‘rare’ as demonstrated by its occurrence for a single couple (Ma, *St*) over the entire set of parameters explored).

Similarly, what sets the specific behaviour shown in figure 8 apart from the others discussed earlier is the uniqueness of its geometrical configuration and its emergence for a single combination of Ma and *St*. In terms of morphology, its hallmark is a clear asymmetry in the size of the lobes, with one lobe being more extended than the other two. We are not in a condition to provide a justification or explanation for this effect, but possible hints or clues can be provided as follows. As it shows up only for the largest value of the Marangoni number considered, it may be the result of two concurrent causes. More precisely, it may be driven by the presence of a second azimuthal wavenumber excited in addition to the primary one (indeed, the corresponding spectrum shown in figure 3*a* indicates that in addition to the multiple *m *= 6, an ‘independent’ mode *m *= 4 with equivalent amplitude is also present) and an effect similar to that used by Kuhlmann & Muldoon [18] to justify 4-lobe structures such as that shown in figure 11 (i.e. the large lobe may be a branch of PAS corresponding to a particular chaotic streamline which is, however, closed by two particle–free-surface collisions). Finally, a certain degree of novelty can also be associated with the PAS shown in figures 12 and 14. Although de-centered structures have often been observed in relation to the mode *m *= 3 and SL-I, to the best of our knowledge, de-centered SL-II configurations have not been reported until now for *m *= 2.

As intermittency, sudden jumps from a solution to another and ‘symmetry breaking’ are the typical features of nonlinear systems governed by the existence of competing attractors, our main conclusion is that this should be taken as a relevant interpretative key for further developments in this field. As an example, an interesting analogy could be implemented with concepts related to the so-called ‘crisis-induced intermittency’, by which an attractor suffers a crisis when two or more attractors cross the boundaries of each other's basin of attraction [44].

## Data Availability

All the required data are contained in the manuscript itself. The data that support the findings of this study are also openly available in the Pure repository of the University of Strathclyde at https://doi.org/10.15129/3a3e3bf2-6b2f-42c5-a875-5048ab0fd9fe.
